# 5′-Nucleotidase Plays a Key Role in Uric Acid Metabolism of *Bombyx mori*

**DOI:** 10.3390/cells10092243

**Published:** 2021-08-30

**Authors:** Linmeng Tang, Dehong Yang, Yaohui Wang, Xu Yang, Kai Chen, Xingyu Luo, Jun Xu, Yujia Liu, Zheng Tang, Qianqian Zhang, Zhiwei Liu, Yongping Huang

**Affiliations:** 1 Key Laboratory of Insect Developmental and Evolutionary Biology, Center for Excellence in Molecular Plant Sciences, Shanghai Institute of Plant Physiology and Ecology, Chinese Academy of Sciences, Shanghai 200032, China; lmtang2016@cemps.ac.cn (L.T.); yangdehong@aliyun.com (D.Y.); wyhhyf@163.com (Y.W.); yangxu@cemps.ac.cn (X.Y.); chenkai2015@sibs.ac.cn (K.C.); xy384@outlook.com (X.L.); xzgxcxj@163.com (J.X.); 18252583552@163.com (Y.L.); 2 CAS Center for Excellence in Biotic Interactions, University of Chinese Academy of Sciences, Beijing 100049, China; 3 Departments of Neonatology, International Peace Maternity and Child Health Hospital of China Welfare Institution, School of Medicine, Shanghai Jiao Tong University, Shanghai 200025, China; Vivian-tang1971@hotmail.com (Z.T.); qianqianzhang1985@hotmail.com (Q.Z.)

**Keywords:** uric acid metabolism, 5′-nucleotidase, ABCG transporter, disease model, *Bombyx mori*

## Abstract

Uric acid (UA) is the end-product in the human purine metabolism pathway. The UA that accumulates in silkworm tissues is excreted as a nitrogen waste product. Here, we first validated that *Bombyx mori* has a homolog of the human gene that encodes the 5′-nucleotidase (5′N) involved in purine metabolism. The *B. mori* gene, *Bm5′N*, is located upstream of other genes involved in UA metabolism in the silkworm. Disruption of *Bm5′N* via the CRISPR/Cas9 system resulted in decreased UA levels in the silkworm epidermis and caused a translucent skin phenotype. When *Bm5′N* mutant silkworms were fed with the uric acid precursor inosine, the UA levels in the epidermis increased significantly. Furthermore, the metabolomic and transcriptomic analyses of *Bm5′N* mutants indicated that loss of the *Bm5′N* affected purine metabolism and the ABC transport pathway. Taken together, these results suggest that the UA pathway is conserved between the silkworm and humans and that the *Bm5′N* gene plays a crucial role in the uric acid metabolism of the silkworm. Thus, the silkworm may be a suitable model for the study of UA metabolism pathways relevant to human disease.

## 1. Introduction

In human and other higher primates, uric acid (UA) is the purine final degradation product. It is synthesized mainly by the liver and excreted by the kidneys [1,2]. Elevated UA is closely associated with metabolic syndromes such as obesity, diabetes, kidney stones, hyperuricemia, and gout [1,3,4,5]. There are both dietary risk factors, such as high-calorie and high purine diets, and genetic risk factors for these diseases [1]. Although multiple genome-wide association studies (GWAS) of global urate disease revealed that at least 28 genes are involved in UA-associated diseases, the molecular mechanisms that underlie these diseases remain unclear [6].

An effective animal model is imperative for studying UA metabolism pathways and expanding knowledge of UA-associated diseases. However, model organisms such as Muridae and Drosophila not only possess a gene encoding *urate oxidase* (*Uro*) but also produce allantoin as the end product of purine metabolites, different from purine metabolism in human [7]. Additionally, reduced *Uro* expression in *Drosophila* and *Muridae* results in a short lifespans and high death rates [1,8,9]. Therefore, these animal models are not appropriate for studying UA disease.

The purine metabolism product in terrestrial insects is UA, which accumulates in insect tissues and is excreted as a nitrogen waste product [10,11]. In *Bombyx mori*, a lepidopteran model insect, UA granules accumulate in larval integument epidermal cells and cause the white skin, which protects against the adverse effects of sunlight [12,13]. Mutations in several genes have been shown to cause translucent larval integument in silkworm and are, thus, implicated in UA metabolism [14]. The proteins encoded by BmXDH (xanthine dehydrogenase), BmMOCO (molybdenum cofactor sulfurase), and BmBLOS2 (biogenesis of lysosome-related organelles complex-1, subunit 2) have functions in purine metabolism similar to homologous proteins in humans [15,16,17,18]. These studies suggest that UA metabolism is conserved between humans and silkworms.

The enzyme 5′-nucleotidase (5′N) is widely distributed in the human body [19]. Mutations in 5′N are correlated with many purine metabolic diseases and abnormalities in UA concentration. Its activity is found in soluble, cytosolic, and membrane-bound forms [20,21]. In the current study, we identified the *B. mori 5′N*. We used the CRISPR/Cas9 system to obtain mutants in which this gene is disrupted. The *Bm5′N* mutants had highly transparent larval integument and significantly reduced UA levels integument (UA accumulation site). When *Bm5′N* mutants were fed human uric acid precursor substances (inosine), the UA content in the epidermis was significantly increased.

Interestingly, a comparison of transcriptomic and metabolomic data for the *Bm5′N* mutant with that of wild-type *B. mori* revealed the involvement of the affected pathway not only in purine nucleotide metabolism but also in lipid metabolism, amino acid metabolism, and energy metabolism, which is consistent with the link between hyperuricemia and gout in human patients [22,23]. The joint analysis showed that the purine metabolites are closely linked to ABC transporter genes. The level of ABCG transporter genes was down regulated in the *Bm5′N* mutant. The ABCG family transporters are involved in human urate excretion [24,25]. Among these down regulated genes, *Bmwh3* and *BmABCG5* have been shown to be involved in UA metabolism in silkworms. These findings strongly indicate that *Bm5′N* is involved in UA metabolism and acts as a rate-limiting enzyme in UA metabolism in the silkworm. Moreover, our data demonstrate that when UA metabolism is altered in humans and silkworms, identical metabolism-related pathways are affected. These findings support the use of the silkworm as a new model for research on human UA metabolism-related diseases.

## 2. Materials and Methods

### 2.1. Silkworm Strain

Silkworms of the Nistari genetic background (a multivoltine, non-diapausing strain) were used in all experiments. Larvae were reared on fresh mulberry leaves under standard conditions, as previously reported [26].

### 2.2. Phylogenetic Analysis

Amino acid sequences of Bm5′N and its homologs were aligned using the CLUSTALX program [27]. Evolutionary analysis and construction of a phylogenetic tree using the neighbor-joining method were performed using the MEGA program Volume 7 [28]. The protein analysis and alignment included 5′N genes from *B. mori* (XP_004932644.1), *Drosophila erecta* (XP_001977658.1), *D. melanogaster* (ACY46082.1), *Homo sapiens* (EAW49656.1), Mus caroli (XP_029328431.1), *Ooceraea biroi* (XP_011330179.1), *Polistes dominula* (XP_015181022.1), *Manduca sexta* (XP_030025513.1), *Danaus plexippus* (XP_032516847.1), and *Chilo suppressalis* (RVE52395.1).

### 2.3. Qualitative and Quantitative Real-Time PCR

Total RNA was isolated from various tissues using the TRIZOL reagent (Invitrogen) according to the manufacturer’s instructions. Samples were treated with DNase I (Invitrogen) to eliminate genomic DNA, and cDNA was synthesized using the ReverAid First Strand cDNA Synthesis Kit (Fermentas). We used SYBR Green Realtime PCR Master Mix (Thermo Fisher Scientific, Waltham, MA, USA) on an Eppendorf Real-time PCR System for Bm5′N qRT-PCR analysis. Samples were pre-incubated at 95 °C for 1 min to denature DNA, followed by 40 cycles of denaturation at 95 °C for 15 s and annealing and an extension at 60 °C for 1 min. Three biological replicates were performed using the *B. mori* ribosomal protein 49 (Bmrp49, 136 bp) as an endogenous control. The primers used for qRT-PCR are listed in Table S1.

### 2.4. Plasmid Construction

Two plasmid construct lines were established for the binary transgenic CRISPR/Cas9 system. One plasmid, pBac [IE1-EGFP-nos-Cas9], reported in our previous studies, used the *B. mori nos* promoter to drive Cas9 nuclease expression together with an IE1 promoter to drive EGFP expression as a selectable marker. We used a second plasmid, pBac [IE1-DsRed2], as a DsRed selectable marker to identify transgenic plasmids expressing Bm5′N single guide RNAs (sgRNAs) [29]. Details of the construction of the guide RNA plasmid and the Cas9 plasmid were described previously [30,31]. A Multi One Step Cloning Kit (Vazyme) was used to assemble the pBac [IE1-DsRed2] plasmid. The primer pairs used in this study are listed in Table S1.

### 2.5. Germline Transformation

The transgenic silkworm strain and the nos-Cas9 line were previously reported [29,30]. We injected U6-sgRNAs and helper plasmids into G0 blastoderm embryos maintained at 25 °C in a humidified environment 10 days prior to hatching. The treated animals were reared to moths on fresh mulberry leaves and crossed with wild-type (WT) moths. After mating the transgenic generation G0 moths with the WT moths, the G1 larvae were screened using fluorescence microscopy (Nikon AZ100), and those that expressed the fluorescent labels were selected. Finally, the U6-sgRNA and nos-Cas9 lines were crossed to provide *Bm5′N* mutants (G2) with double fluorescence, which were used in subsequent experiments.

### 2.6. CRISPR/Cas9 Genome Editing

SgRNAs were designed according to the dual sgRNAs strategy, as previously described [32]. SgRNAs were synthesized through an invitrogen High Yield Transcription Kit (00945070). Cas9 protein was purchased from Thermofisher (A36498). We mixed 1 ug of Cas9 protein and 300 ng/uL sgRNAs in a 5 uL volume for injection [32].

### 2.7. Mutagenesis Analysis

Genomic DNA of mutated animals was extracted at the 5th instar larval stage using standard SDS lysis-phenol treatment after incubation with proteinase K, followed by RNase treatment and ethanol precipitation. Mutation events were detected by PCR amplification using gene-specific primers bound upstream or downstream from each target (Table S1), and amplified products were visualized using agarose gel electrophoresis. Amplicons were sub-cloned into the pJET-1.2 vector (Fermentas), and clones of each line were selected and sequenced using an Illumina NextSeq 500 platform (Sunnybio).

### 2.8. Quantification of UA Content

UA was extracted from various tissues at the fifth larval instar. The excised samples were washed in PBS buffer. After a 10-h drying process, samples were boiled in deionized water for 30 min. The absorbance of the resulting liquid at 510 nm was then measured with a C012-2-1 Uric Acid Kit (Nanjing Jiancheng Bioengineering Institute) using a multi-mode microplate reader (Varioskan Flash) [33].

### 2.9. Feeding Experiments

The fifth-instar larvae were divided into three groups, each group has fifteen animals. Each larva was fed with a 5 cm^2^ piece of a mulberry leaf to which 100 μL of 20 mg/mL inosine had been applied to each. Controls were fed with a leaf wiped with the same volume of water. Feeding was started from the first day of the fifth instar and continued for 4 days.

### 2.10. LC-MS Sample Preparation and Experiments

To investigate metabolic changes, we used mass spectrometry imaging to detect compounds produced by Bm5′N mutants and WT animals (6 animals per group). Six replicates of each tissue were analyzed. We used a ratio of 25 mg of each tissue to 800 μL pre-cooled extraction reagents (methanol:acetonitrile:water, 2:2:1, *v*/*v*/*v*), and stored extracts at 4 °C until analysis. We controlled sample preparation quality with internal standards mix 1 (IS1) and internal standards mix 2 (IS2). IS1 contained d-, l-methionine (100 ppm, TRC), phenylalanine (100 ppm, CIL), d-, l-alanine (100 ppm, TRC), l-threonine (100 ppm, CIL), l-aspartic acid (100 ppm, TRC), d-, l-2-aminobutyric acid (100 ppm, TRC), l-arginine (100 ppm, CIL). IS2 was purchased from Avanti (SPLASHTM Lipidomix Mass Spec Standard, 330707) and contains LPC, LPE, PI, PA, SM, cholesterol, CE, PC, PE, PG, PS, MG, DG, and TG. After homogenizing for 5 min using a TissueLyser (JXFSTPRP), we sonicated samples for 10 min followed by incubation at −20 °C for 1 h, centrifuged at 4 °C for 15 min at 25,000 rpm, and freeze-dried the supernatant. We then resuspended metabolites in 300 μL of 10% methanol and sonicated them at 4 °C for 10 min, followed by centrifugation for 20 min at 25,000 rpm. Supernatants were analyzed on a Waters 2D UPLC interfaced with a Q Exactive high-resolution mass spectrometer (Thermo Fisher Scientific). LC-MS experiments were performed by Beijing Genomics Institute, Shenzhen, China as previously described [34,35].

### 2.11. Metabolomics Data Analysis

We analyzed raw data files using Progenesis QI software Volume 2.1. We then evaluated the models with the relevant R2 and Q2 as described elsewhere [36] and used Experiment Viewer (MeV) Volume 4.9 software to draw heatmaps. We identified candidate metabolites with calculations in both positive and negative ion modes. We performed metabolite pathway analysis based on the Kyoto Encyclopedia of Genes and Genomes (KEGG) pathway databases and The Human Metabolome Database [37,38].

### 2.12. RNA Sequencing and Analysis

Three samples from WT and three from Bm5′N mutants were sequenced by BGI using BGISEQ-500. All the raw reads data were filtered to remove adaptor-based reads and lower quality sequences [39]. The transcripts were annotated using BLAST against the NCBI database. The Bombyx mori reference genome version was GCF_000151625.1_ASM15162v1. Gene ontology (GO) functional classification was performed through Blast2GO [40]. The biological functions of the differentially expressed genes were obtained from the KEGG pathway database.

### 2.13. The Metabolites and Genes Correlation Analysis

The correlation of differential genes and differential metabolites was analyzed according to the Canonical Correlation Analysis (rCCA) and mixOmics [41]. To measure the association between genes and metabolites, correlation analysis (RCCA) and the block. splsda function in a mixOmics package were adopted. The results were visualized using the function of plotVar and circosPlot [42].

### 2.14. Statistical Analysis

All experiments in this study were performed with at least three biological replicates, and error bars denote means ± SEM. A two-tailed Student’s *t*-test was used to evaluate the differences between WT and mutant individuals. Asterisks mark statistically significant differences.

## 3. Results

### 3.1. Phylogenetic Identification of Bm5′N

**Figure 1:**
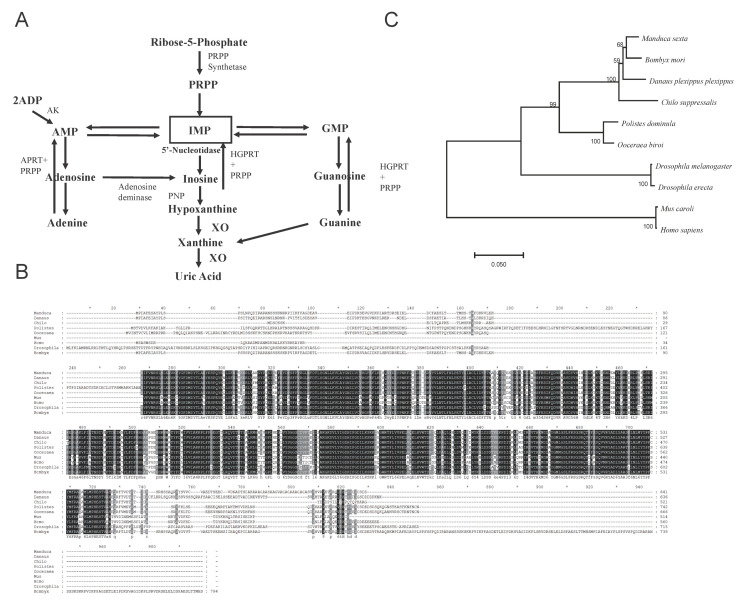
Uric acid metabolism pathway genes in human and silkworm. (**A**) The uric acid metabolism pathway in humans. (**B**) Phylogenetic tree of 5′N proteins. (**C**) Alignment of 5′N proteins.

**Figure 2:**
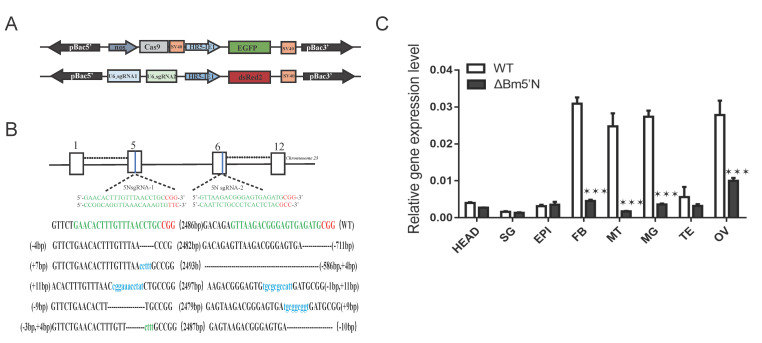
Construction of ΔBm5′N mutants using the CRISPR/Cas9 genome editing system (**A**) Constructs for piggyBac-based transgenic transformation. Expression of Cas9 is driven by a nos promoter with EGFP as a fluorescent marker. Expression of the two Bm5′N sgRNAs under the control of U6 promoter is indicated by DsRed2. (**B**) *Bm5′N* gene structure and sgRNA-target sites. Boxes denote the 13 exons of this gene. Bm5′N-specific sgRNA target sites are located in exons five and six. The sgRNA-targeting sequences are in green, and the adjacent protospacer motif (PAM) sequences are in red. The deletion mutations in *Bm5′N* in selected mutants are shown. (**C**) Relative *Bm5′N* mRNA expression in eight tissues of ΔBm5N mutant and WT. Abbreviations: head (HEAD), silk gland (SG), epidermis (EPI), fat body (FB), Malpighian tubule (MT), testis (TE), ovary (OV). Three biological replicates were performed; error bars denote ± SD. The asterisks (***) indicate significant differences relative to WT (*p* < 0.001, *t*-test).

To identify the UA metabolism genes in the silkworm, we compared the sequences of genes in the human UA metabolism pathway with the published silkworm genome [43]. We cloned silkworm homologs by designing primers to amplify their coding sequences (Figure 1A and Table S2). By analyzing the amino acid sequences of putative purine metabolism genes, we identified the Bm5′N alignment of the 5′N amino acid sequences of human, mouse, and insect species (Figure 1B,C) indicated that 5′N is highly conserved, suggesting that it has retained a similar function in purine metabolism during evolution. QRT-PCR analysis showed that *Bm5′N* was expressed in various tissues and that it was expressed at high levels in malpighian tubules and fat body (Figure 2C).

### 3.2. Targeted Mutagenesis Using the CRISPR/Cas9 System

To investigate the function of *Bm5′N* in vivo, we obtained *Bm5′N* mutants using the CRISPR/Cas9 approach as previously described [44]. Through germline transformation, we generated two independent transposon-mediated transgenic lines (Figure 2A). We screened these two lines with an EGFP fluorescent marker for the Cas9-expressing line and a DsRed2 fluorescent marker for the sgRNA-expressing line. After crossing these two lines, offspring exhibiting fluorescence markers for both Cas9 and sgRNA expressions were detected. PCR sequencing demonstrated that these individuals carried large deletions of the *Bm5′N* gene (Figure 2B).

### 3.3. Loss Function of Bm5′N Results in Translucency of the Larval Integument

**Figure 3:**
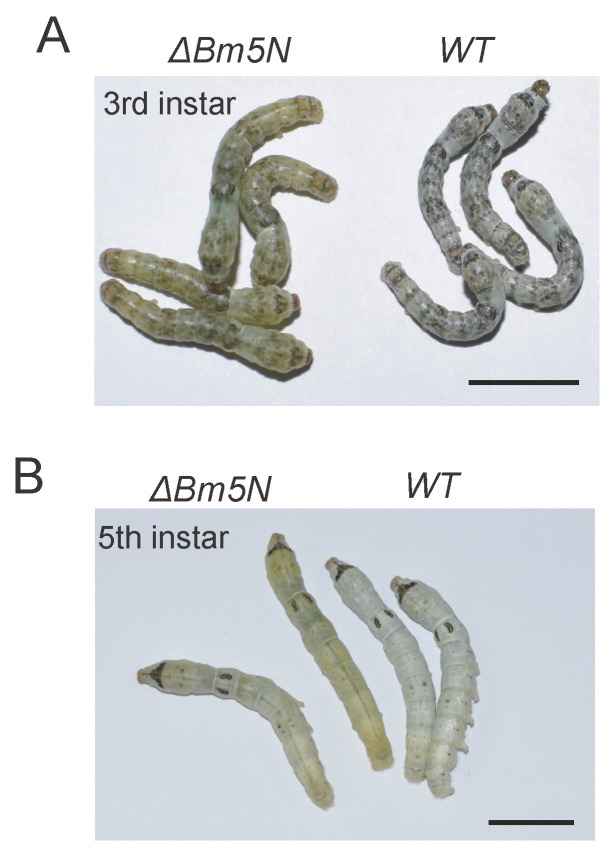
*Bm5′N* mutation results in the transparency of the larval integument. (**A**) Photographs of WT and *ΔBm5′N* G2 larva at the third instar. (**B**) Photographs of WT and mutant G2 larva at the fifth instar. Bars = 1 cm.

**Figure 4:**
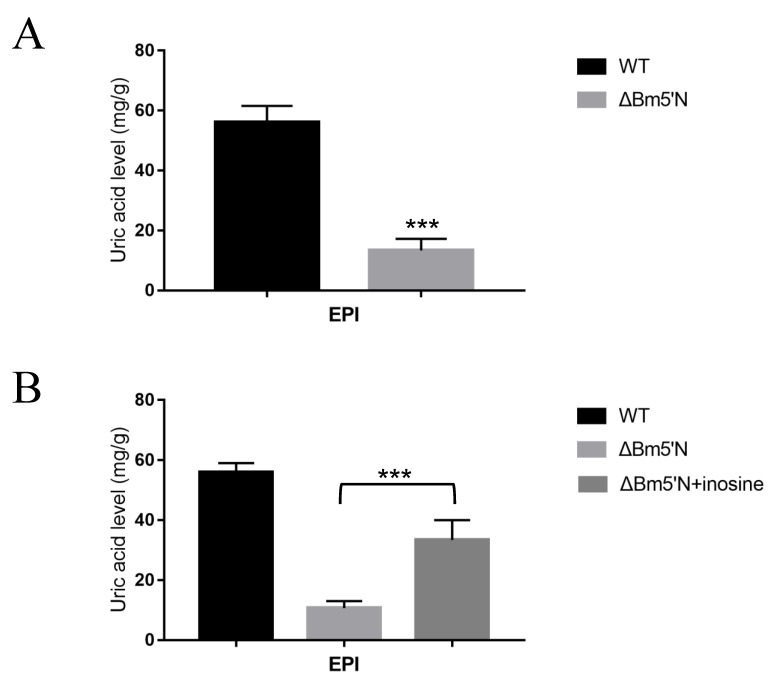
*Bm5′N* loss function decreases UA content. (**A**) UA concentration in the integument of WT and *ΔBm5′N* epidermis (EPI) of fifth instar larvae. (**B**) UA concentrations in fifth instar larvae fed UA precursors. Asterisks indicate significant differences with a two-tailed *t*-test: *** *p* < 0.001.

In the silkworm, UA is synthesized in the fat body and stored in the epidermis [45]. It is the UA accumulation in cells of the larval integument that causes silkworms to appear opaque and white [12,13]. We observed that the *Bm5′N* mutants had highly transparent larval integument from the second to the fifth instar, compared with WT animals (Figure 3A,B), indicating that the loss of the *Bm5′N* gene affected UA metabolism. These results are supported by a recent independent study, also revealing that *Bm5′N* is an essential gene in the p-oily (op) mutant in the silkworm [46]. The measurement of the UA content reduction in the integument of *Bm5′N* mutants compared to WT which can be seen in Figure 4A. These results demonstrate that the *Bm5′N* gene is essential for UA synthesis in the silkworm.

### 3.4. Feeding Experiments Indicate That Bm5′N Is Necessary for UA Biosynthesis

In humans, 5′N is involved in the synthesis of UA precursors (Figure 1A) [47]. We speculated that *Bm5′N* had a similar function in the silkworm. To investigate whether *Bm5′N* participates in UA precursor synthesis, we performed feeding experiments with inosine supplementation using fifth-instar larvae. We collected the epidermis for UA testing. Supplementation caused a significant increase in UA content in the epidermis of mutants (Figure 4B). These results indicate that the *Bm5′N* gene is involved in the synthesis of UA precursor substances, which means that the *Bm5′N* function is conserved between humans and silkworm.

### 3.5. Bm5′N Mutant and WT Metabolite Profiles Differ in Purine Metabolism

**Figure 5:**
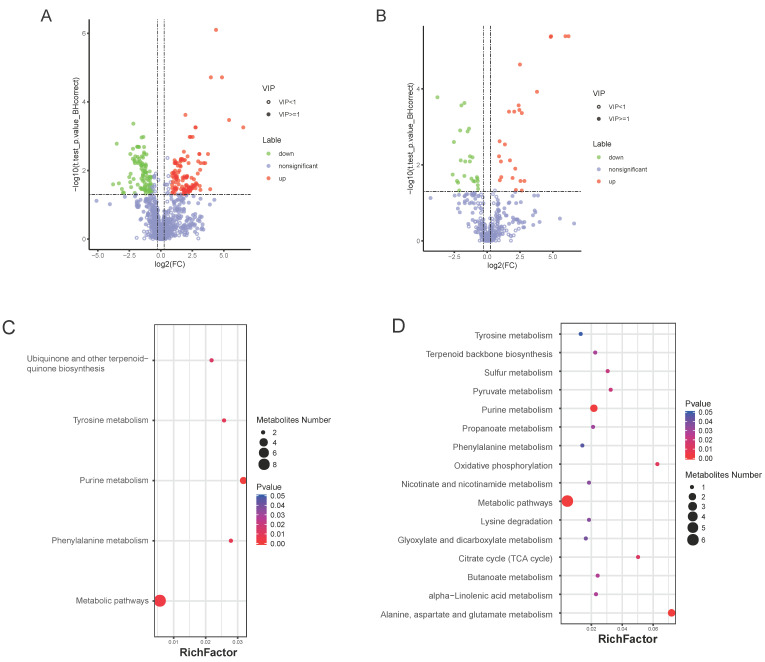
Metabolite production is disrupted in *Bm5′N* mutant. (**A**,**B**) Volcano plot of metabolites differentially expressed in malpighian tubules of WT and *ΔBm5′N* in (**A**) positive ion mode and (**B**) negative ion mode. Green, down-regulated metabolites; red, up-regulated metabolites; purple, no significant difference in metabolites. Differentially produced metabolites were identified by the following criteria: *p*-value < 0.05; Variable Important for the Projection ≥ 1; Fold-Change ≥ 1.2 or Fold-Change ≤ 0.83. (**C**,**D**) Enrichment factors representing the differential proportion of a metabolite relative to all metabolites in this pathway for differentially produced metabolites detected in (**C**) positive ion mode and (**D**) negative ion mode. The dot size indicates the numbers of metabolites.

The malpighian tubule is the most important UA excretory organ in insects [48]. Thus, to further investigate the role of *Bm5′N* in UA metabolism, we examined metabolites in the malpighian tubules of the *Bm5′N* mutant and WT using ultraperformance liquid chromatography coupled with Q-TOF mass spectrometry. We classified all detected metabolites and assessed the differences between the *Bm5′N* mutant and WT. We identified a total of 1293 and 415 compounds in samples with the electrospray ionization in positive and negative modes (Figures S1, S2 and S3). The ion chromatograms exhibited a stable retention time without apparent peak drifts. Using cluster analysis, we identified 225 metabolites present at different concentrations in mutant and WT malpighian tubules in the positive ion mode and 49 differential metabolites in the ESI-mode (Figure 5A,B and Figure S4). Pathways were identified using enrichment analysis of the metabolites present in different amounts. The results showed significant enrichment of sixteen KEGG pathways including purine nucleotide metabolism, lipid metabolism, amino acid metabolism, and energy metabolism (Figure 5C,D). These data also indicated that the *Bm5′N* gene is involved in purine metabolism, consistent with previous results in humans [49].

### 3.6. Level of ABCG Transporter Genes Were Down Regulated in Bm5′N Mutant

**Figure 6:**
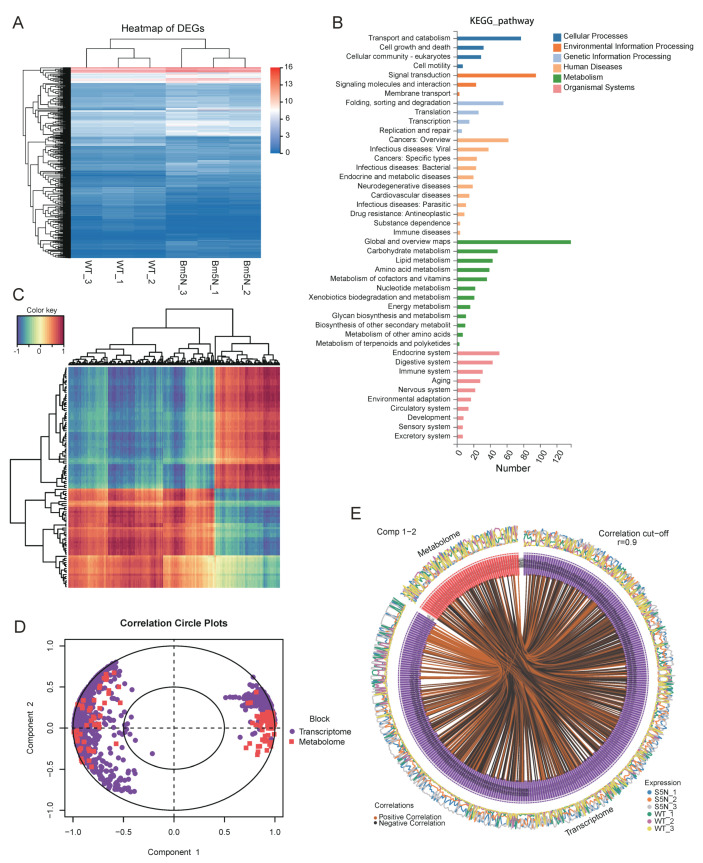
*Bm5′N* loss alters metabolome and transcriptome. (**A**) Heat map of differential gene expression detected in transcriptomics analysis. Statistical analysis (|fold change| ≥ 3 and *p*-value <0.05) identified 972 genes. (**B**) Column chart of the KEGG enrichment results of DEGs. (**C**) Correlation cluster heat map of metabolomics and transcriptomics. Each row represents a differentially produced metabolite and each column represents a differentially expressed gene. Red indicates positive correlation, whereas blue indicates negative correlation. (**D**) Concentric circle of differentially expressed genes and metabolites. The RCCA results were visualized using plotVar. Every dot represents one gene, each square represents a metabolite. An angle <90° indicates a positive correlation, >90° means a negative correlation. Taking the center of the circle as the starting point, the longer line length means a stronger relationship. The variables far away from the center of the circle have a strong correlation. (**E**) Circus analysis of differential genes and differential metabolites. The RCCA results were visualized using circosPlot. The line of circle represents the correlation coefficients were larger than 0.9 between differential genes and differential metabolites. Blue curves of peripheral regions represent the expression of differential metabolites. Orange curves of peripheral regions represent the expression of differential genes.

To identify genes that are differentially expressed in *Bm5′N* mutant and WT, we performed transcriptomic analyses of malpighian tubule tissue of the *Bm5′N* mutant and WT. Transcriptome data identified 3763 candidate genes. Additionally, a total of 972 differentially expressed genes were detected. We conducted pathway enrichment analysis and cluster analysis of differentially expressed genes (DEGs) bases on the KEGG database (Figure 6A,B). A large number of DEGs were involved in metabolic pathways. Interestingly, lots of human disease-associated pathways were related to these DEGs. To verify the consistency of results between metabolomics and transcriptomics data, we analyzed the correlation between DEGs and differentially produced metabolites through cluster analysis and mixOmics analysis. The RCCA results were visualized by the function of plotVar and circosPlot (Figure 6D,E). Overall, robust associations between several DEGs and differential metabolites were presented by using different methods, which further confirmed robust correlations between DEGs and differential metabolites. (Figure 6C–E). Our results suggest that the mutation of the *Bm5′N* gene resulted in the changes of metabolites and genes in the silkworm. This provides support for upstream gene research.

**Figure 7:**
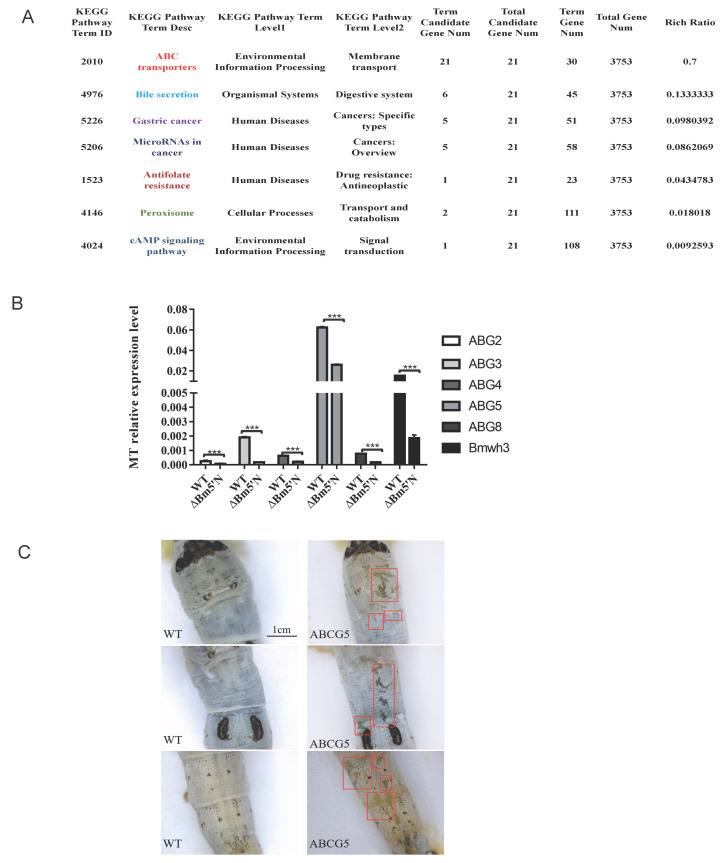
Transporter expression is disrupted in *ΔBm5′N* larvae. (**A**) Transporter genes differentially expressed in *ΔBm5′N* and WT larvae. (**B**) qRT-PCR analysis of ABC related gene’s mRNA expression in *ΔBm5′N* and WT at the fifth larval instar MT. (**C**) Enlargement of the translucent mosaic larval of *ΔBmABCG5* at the fifth instar. The red box indicated representative areas of the translucent integument. *** *p* < 0.001.

The joint analysis showed that the purine metabolites are closely linked to the ABC transporter gene. Moreover, the transporter pathway is the main pathway enriched among DEGs (Figure 7A), and thus we analyzed those transporter genes. To verify the results of RNA-seq analyses, we measured the expression level of *ABG2*, *ABG3*, *ABG4*, *ABG5*, *ABG8*, and *Bmwh3* through qRT-PCR. The mRNA levels of these genes were significantly down-regulated, especially in the malpighian tubule (Figure 7B). Interestingly, *Bmwh3* had been demonstrated to be involved in UA epidermis transport in the silkworm previously [45,50]. To test whether these genes are involved in uric acid metabolism, we chose the highest expression gene *ABG5*(*BGIBMGA002712*) for the gene-editing experiment (Figure S6). As expected, we obtained *ΔBmABCG5* larvae that had translucent integument (Figure 7C). Taken together, these results suggest that the *Bm5′N* gene is an upstream gene in purine metabolism.

## 4. Discussion

In this study, we demonstrated that the *Bm5′N* gene is involved in UA metabolism in the silkworm. The 5′N enzyme hydrolyzes nucleoside monophosphates or deoxynucleotide monophosphates to nucleotides in humans [51]. In humans, the enzyme is broadly expressed in tissues such as the skeleton, muscle, brain, testis, liver, colon, and stomach [51]. Our results showed that the *Bm5′N* gene is expressed in many silkworm tissues with high levels in malpighian tubules and fat body. The malpighian tubules and fat body in insects are sites of purine metabolism and are considered equivalent to the human kidney and liver. Mutation of the *Bm5′N* gene caused high transparency of the larval integument, where UA accumulates, indicating that the *Bm5′N* gene is necessary for UA production. Recently, two silkworm genes, *BmMoco* and *BmXDH*, homologous to genes in humans, have been shown to encode proteins necessary for the syntheses of xanthine and hypoxanthine [17,52]. Our data suggest that *Bm5′N* acts upstream of *BmMoco* and *BmXDH* because the adverse effects of the deletion of *Bm5′N* on UA production are relieved by feeding larvae UA precursors inosine. This is consistent with the function of 5′N in the corresponding human metabolic pathway (Figure 1).

The natural mutant *op* exhibited translucent larval integument, mortality, and male infertility [46]. The *Bm5′N* gene is responsible for the *op* locus [53]. We found that most *Bm5′N* mutants were lethal at the pupal stage. We thought that the gene-knockdown effects work better in the binary transgenic CRISPR/Cas9 system.

Alanine, aspartate, glutamate metabolism, citrate cycle metabolism, unsaturated lipid metabolism, and other metabolic pathways were disrupted in hyperuricemia and gout patients [22,23]. In our metabolite analysis, we also found these metabolic pathways were disrupted by the *Bm5′N* deletion. The additional metabolites identified in the present study will further enhance our understanding of uric acid metabolism in the silkworm.

Our analyses revealed that the UA synthesis pathway is conserved between humans and silkworms. However, patients with hypouricemia and gout may also have abnormalities in uric acid transport and secretion in kidney and kidney tubules [54,55,56]. This led us to evaluate the metabolome and transcriptome of the malpighian tubule in the *Bm5′N* mutant. Our RNA-seq results indicate that ABC family transporter gene expression is disrupted by the loss of *Bm5′N*. The ABC family transporters are involved in human urate excretion [24,25]. Our joint metabolome/transcriptome analysis showed that purine metabolites are tightly linked with the ABC transporter gene expression. This phenomenon has been described previously in *w-3*, *od*, *oa*, and *otm* silkworm mutants, but the underlying mechanism is unknown [14]. The expression level of *ABG2, ABG3, ABG4, ABG5, ABG8,* and *Bmwh3* were significantly down-regulated in *ΔBm5′N*. Among these down regulated genes, we obtained *ΔBmABCG5* larval that had translucent integument. Interestingly, *Bmwh3* had translucent integument that had been demonstrated previously [45,50]. These results provide a direction for our next work.

Our data indicate that UA metabolism and UA excretion systems are similar in humans and silkworms. Nevertheless, for a deeper understanding of the complete function of the UA metabolism, it is vital to investigate gene functions along with the entire UA metabolic network in the silkworm. Such research will shed light on the roles of homologous genes in humans with metabolic conditions as hyperuricemia and gout and on the effects of drugs used to treat these conditions, such as the xanthine dehydrogenase inhibitor drug allopurinol, which is widely used to prevent UA production. Considering its short life cycle, ease of mass rearing, and the availability of a genetic tool kit, further investigation of the UA metabolism genes in the silkworm could provide a powerful model to study UA metabolism and UA-associated disease in humans [57,58,59].

## Data Availability

The data presented in this study are available within this article.

## Supplementary Materials

The following are available online at https://www.mdpi.com/article/10.3390/cells10092243/s1, Figure S1: Proteins identified in malpighian tubule samples in the (**A**) positive ion mode and (**B**) negative ion mode. y-axis, intensity, Figure S2: Principal component analysis of proteins differentially expressed in malpighian tubule samples identified in (**A**) positive ion mode and (**B**) negative ion mode, Figure S3: Classification of metabolites. (**A**) Classification of metabolites in positive ion mode. (**B**) Classification of metabolites in negative ion mode. x-axis, number of metabolites; y-axis, KEGG pathway annotations, Figure S4: Hierarchical cluster analyses of the metabolite distribution in ΔBm5′N mutant and WT of metabolites detected in (**A**) positive ion mode and (**B**) negative ion mode, Figure S5: CRISPR/Cas9 induced mutagenesis of Bm5′N mutant, Figure S6: CRISPR/Cas9 induced mutagenesis of BmABCG5 mutant, Table S1: Primers used in this work, Table S2: Silkworm homologous gene ID.
