# Supplementary material for: 5′-Nucleotidase Plays a Key Role in Uric Acid Metabolism of Bombyx mori
Source: Cells. 2021 Aug 30;10(9):2243. doi: 10.3390/cells10092243 (PMC8468349; doi:10.3390/cells10092243)
Supplement: Supplementary file 1 [file cells-10-02243-s001.zip › supplemental.pdf]

Supplemental

Supplemental Figures

Figure S1. Proteins identified in malpighian tubule samples in the (A) positive ion mode and (B) negative ion mode. y-axis, intensity.

(A)

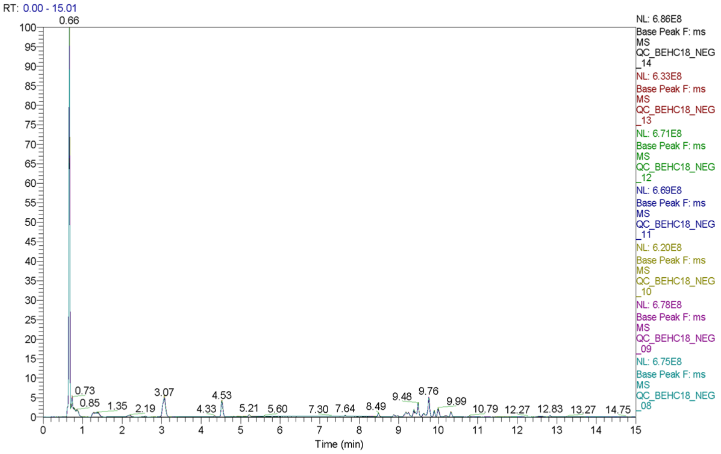

(B)

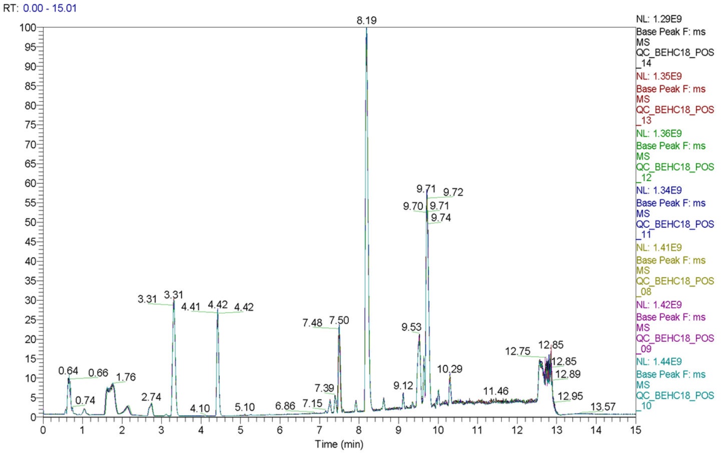

Figure S2. Principal component analysis of proteins differentially expressed in malpighian tubule samples identified in (A) positive ion mode and (B) negative ion mode.

(A)

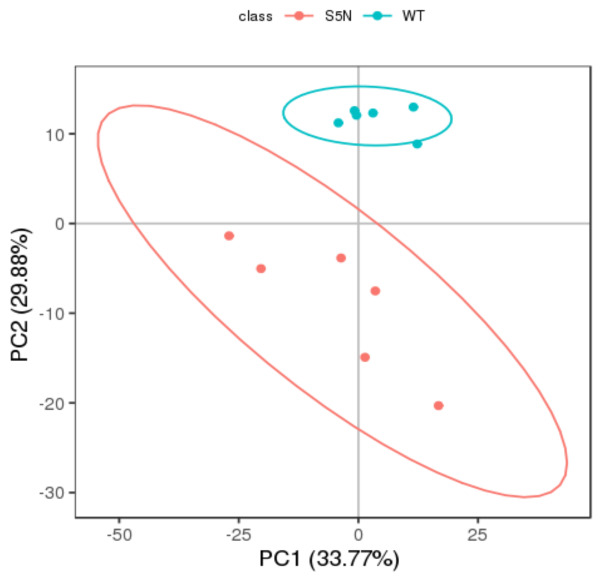

(B)

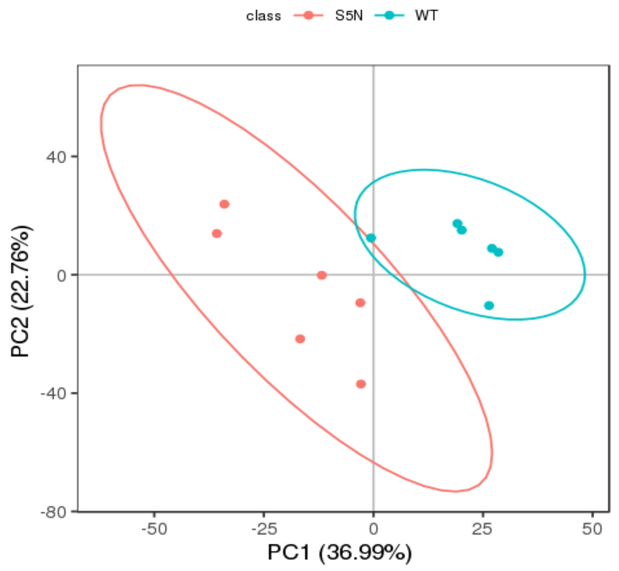

Figure S3. Classification of metabolites. (A) Classification of metabolites in positive ion mode. (B) Classification of metabolites in negative ion mode. x-axis, number of metabolites; y-axis, KEGG pathway annotations.

(A)

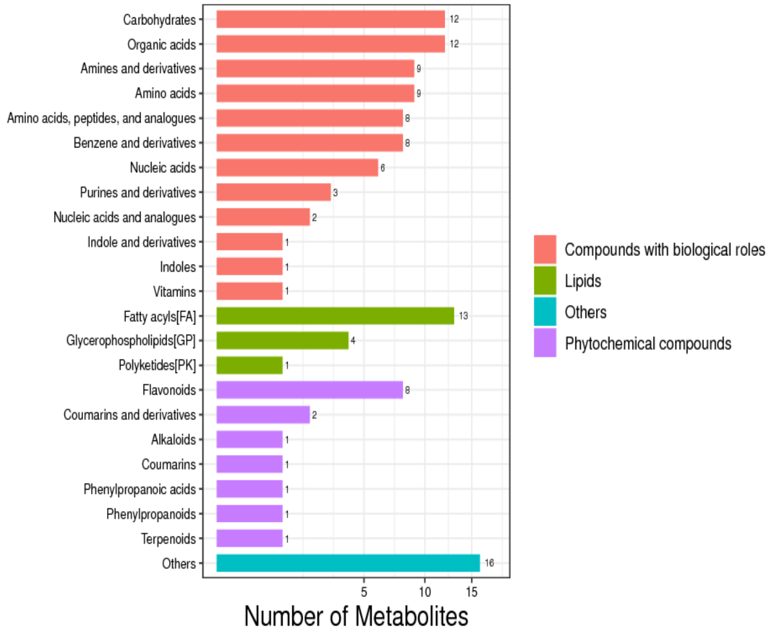

(B)

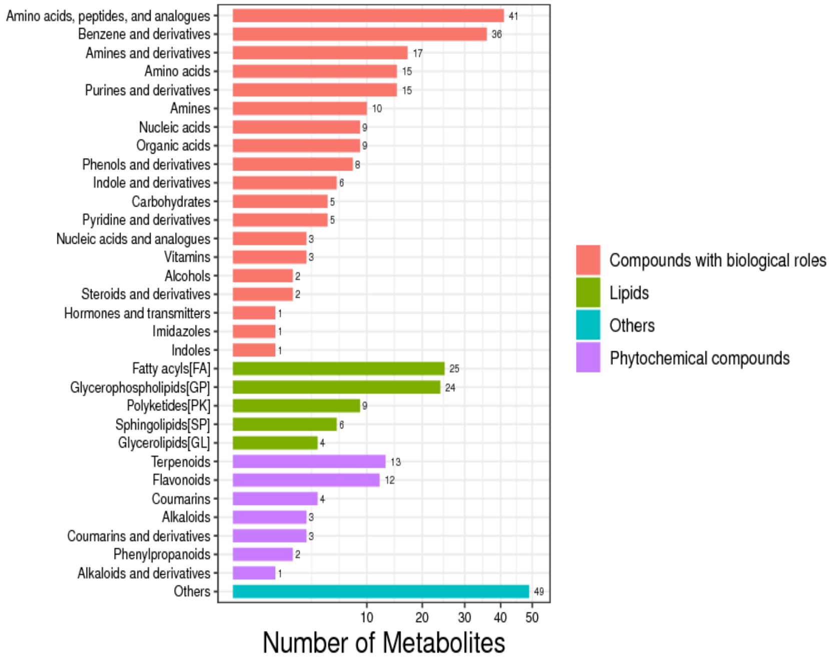

Figure S4. Hierarchical cluster analyses of the metabolite distribution in ΔBm5'N mutant and WT of metabolites detected in (A) positive ion mode and (B) negative ion mode. Each column represents an individual, and each row represents a metabolite. The color (from green to red) represents metabolite expression intensity from low to high.

(A)

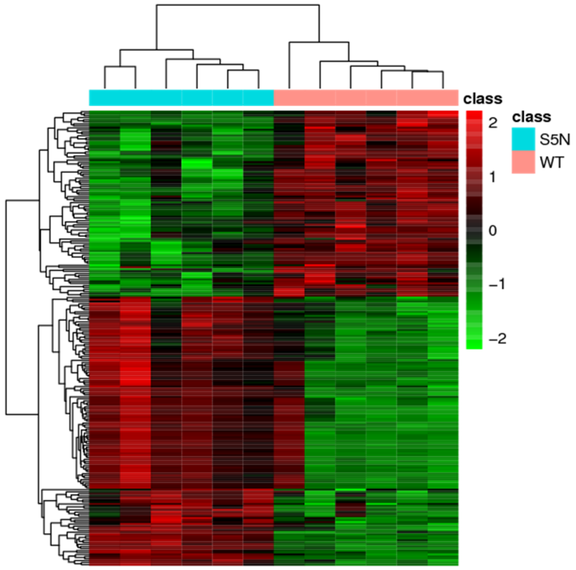

(B)

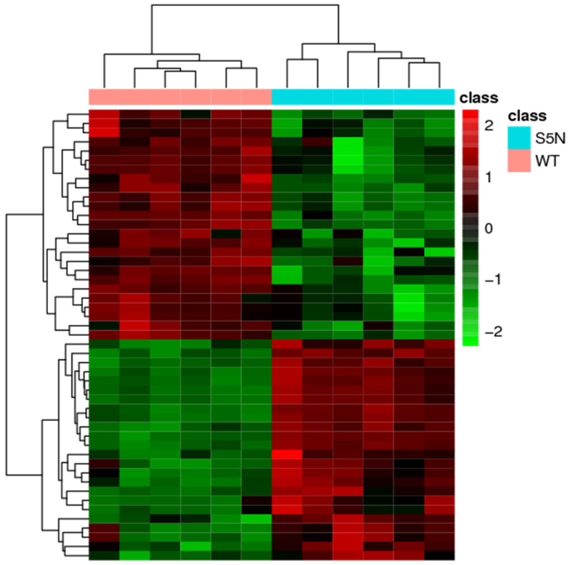

Figure S5. CRISPR/Cas9 induced mutagenesis of Bm5'N mutant. Target sites are shown as red squares. The deletion mutations were detected in the five mutant samples.

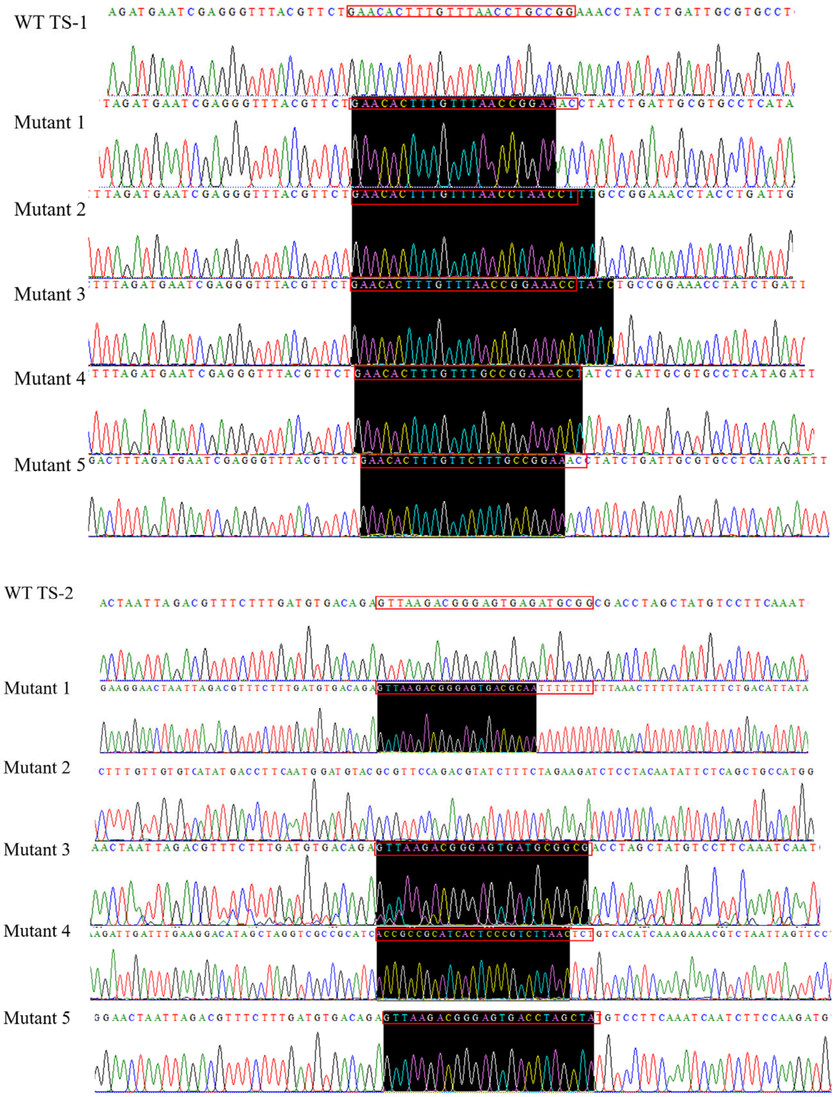

Figure S6. CRISPR/Cas9 induced mutagenesis of BmABCG5 mutant. Target sites are shown as red squares. The deletion mutations were detected in the five mutant samples.

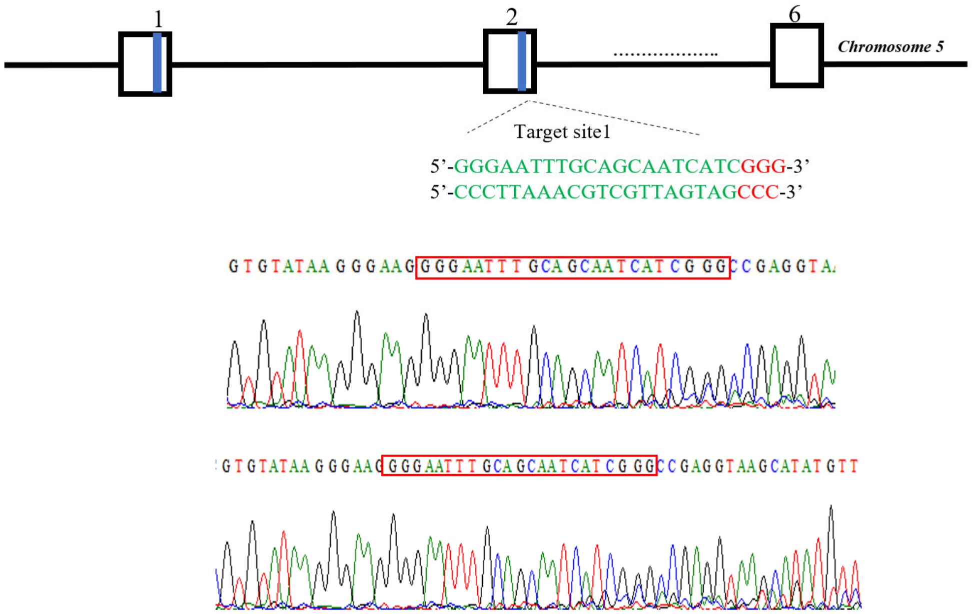

Table S1 Primers used in this work

Primer name	Primer sequence(5'to3')	The purpose
5N-CDS-F	ATGGACACCACTAGAATAATGC	CDS Clone
5N-CDS-R	TTATTTGTGTTGCGCCGCCTT	CDS Clone
5N-qPCR-F	CGATGCCTACGGGAATATTTTAGTG	qRT-PCR analysis
5N-qPCR-R	ATCGCCGTGTATGTGAACGT	qRT-PCR analysis
5N-TS1-F	AGTGCAACTTTAACTAAGTGAC	Identification of mutations
5N-TS1-R	ACGAGTACCATCCAGAGACT	Identification of mutations
5N-TS2-F	CTTTGTTGTGTCATATGACCTT	Identification of mutations
5N-TS2-R	AGAGTGGTTCCCTCGCCGAAG	Identification of mutations
BmRP49-F	TCAATCGGATCGCTATGACA	qRT-PCR analysis
BmRP49-R	ATGACGGGTCTTCTTGTTGG	qRT-PCR analysis
wh3-qPCR-F	AAATCGCAACTACGGAGCCA	qRT-PCR analysis
wh3-qPCR-R	CATGATGGCCAGCAGTTCAC	qRT-PCR analysis
AB2-qPCR-F	TGTCGCCGCTAAACTAAAACT	qRT-PCR analysis
AB2-qPCR-R	ACTGTATCTAGTCCGCTAGTTGG	qRT-PCR analysis
AB3-qPCR-F	CACTGGGTCTGTACGAGCAT	qRT-PCR analysis
AB3-qPCR-R	AGTTCTTTGTGGTGCCCTGG	qRT-PCR analysis
AB4-qPCR-F	ACGAAGCAATGTCTCTCGCA	qRT-PCR analysis
AB4-qPCR-R	CCAGGAACATGAGGGATGGA	qRT-PCR analysis
AB5-qPCR-F	TCTAGTGTCGTTAGCCGGGA	qRT-PCR analysis
AB5-qPCR-R	ATTGATTTGTTCGGTTGCTTCGT	qRT-PCR analysis

AB8-qPCR-F	AGGTTGCGAACAAATGCGTC	qRT-PCR analysis
AB8-qPCR-R	TCCATTGTGAGTATGATAGCGGT	qRT-PCR analysis
KpnI-F	CGAGGTCGACGGTATCGATAAGGTTATGTAGTACAC ATTGTTGTA	Plasmid construction
U6-5NTS1-R	GCAGGTTAAACAAAGTGTTCACTTGTAGAGCACGAT ATTTTGTAT	Plasmid construction
U6-5NTS1-F	GAACACTTTGTTTAACCTGCGTTTTAGAGCTAGAAAT AGCAAGTT	Plasmid construction
overlap-R	CCGCGGAGTCAATGGCTAGCAAAAAAGCACCGACT CGGTG	Plasmid construction
overlap-F	GCTAGCCATTGACTCCGCGGAGGTTATGTAGTACAC ATTG	Plasmid construction
U6-5NTS2-R	CATCTCACTCCCGTCTTAACACTTGTAGAGCACGATA TTTTGTAT	Plasmid construction
U6-5NTS2-F	GTTAAGACGGGAGTGAGATGGTTTTAGAGCTAGAAA TAGCAAGTT	Plasmid construction
HindIII-R	TTTTCTTGTTATAGATATCAAAAAAAGCACCGACTCG GTG	Plasmid construction
AD-CDS-F	ATGCTTCAGCTTCAAAGGTATTATGT	CDS Clone
AD-CDS-R	TCTTTCCAATGATTAACTCTCTAATAT	CDS Clone
AK-CDS-F	ATGGACGTTTCTGATTCCATATGT	CDS Clone
AK-CDS-R	TCAGTCATTGTATTCGCTGGGTC	CDS Clone
AMPD1b-CDS-F	ATGTTCGAGGTCCCGGCGACAG	CDS Clone
AMPD1b-CDS-R	TACGTACGCTAAACAAGTTGTCCAG	CDS Clone
ART1-CDS-F	ATGTCCCAAACAACGATTACATCAT	CDS Clone
ART1-CDS-R	CAGCCAAAGCTTCTAATTTACAG	CDS Clone
PNP-CDS-F	ATGGCACCTATAAACGCGAACGATAT	CDS Clone
PNP-CDS-R	TCATGGTGCATCAGGGTCGGCCT	CDS Clone
PRPS1b-CDS-F	ATGCCGAACATAAAAGTGTTTACTGG	CDS Clone
PRPS1b-CDS-R	TTAATATGGCACGTTAGTAAAAAGATAC	CDS Clone
PRPS1-CDS-F	ATGTCTGTGCACAAGTCGAACCA	CDS Clone
PRPS1b-CDS-R	TTAATATGGCACGTTAGTAAAAAGATAC	CDS Clone
Xan-CDS-F	ATGAAATCTAGTAATCAGATCAAC	CDS Clone
Xan-CDS-R	TTGCACCTATAATTGAGTTGACAT	CDS Clone
XDH-CDS-F	TAACGATAAACATTACACTGTGGAC	CDS Clone
XDH-CDS-R	CGGTGAATGGCAGATCGATATCT	CDS Clone
Xan2-CDS-F	ATGGTCATTTGTCTCGTTCACATC	CDS Clone
Xan2-CDS-R	GCTTCCTATCCTGGGTGAAATC	CDS Clone
ABCG5-sg1	TAATACGACTCACTATAGGGAATTTGCAGCAATCATC GTTTTAGAGCTAGAAATAGCAAGTTAAAATAAGGCT AGTCCGTTATCAACTTGAAAAAGTGGCACCGAGTCG GTGCTTTT	Gene editing

AMPD1-F	ATGTGTTTAATTGCAGGCAGTGAAAG	CDS Clone
AMPD1-R	CTAAGTGGTAGGTTGAGCTTT	CDS Clone

Table S2 Silkworm homologous gene ID

Human gene	Silkworm homologous gene
phosphoribosyl pyrophosphate synthetase 1(PRPS1)	BGIBMGA001386
(PRPS1b)	BGIBMGA001570
adenosine monophosphate deaminase 1（AMPD1）	BGIBMGA000806
(AMPD1b)	BGIBMGA000807
adenine phosphoribosyl transferase（ART1）	BGIBMGA011818
adenosine kinase（AK）	BGIBMGA007418
adenosine deaminase(AD)	BGIBMGA001278
purine nucleoside phosphorylase(PNP)	BGIBMGA011774
Xanthine Dehydrogenase(Xan)	BGIBMGA008244
（Xan2）	BGIBMGA008421
5’-nucleotidase（5N）	BGIBMGA011479
XDH xanthine dehydrogenase(XDH)	BGIBMGA008439
